# The effect of exogenous ketone bodies on cognition across health and disease: a systematic review and meta-analysis

**DOI:** 10.3389/fnut.2026.1802531

**Published:** 2026-04-15

**Authors:** Bruno Bonnechère, Elizabeth B. Stephens, Amy C. Boileau, Martin Ducker, Brianna J. Stubbs

**Affiliations:** 1 Faculty of Rehabilitation Science, University of Hasselt, Hasselt, Belgium; 2 Buck Institute for Research on Aging, Novato, CA, United States; 3 Component Health Ltd., Dublin, Ireland

**Keywords:** cognition, D-*β*-hydroxybutyrate, ketone ester, ketones, meta-analysis

## Abstract

**Introduction:**

Cognitive function is closely linked to brain energy metabolism and may be compromised by aging, metabolic stress, and neuropsychiatric disease. Ketone bodies can serve as an alternative cerebral fuel and may also exert signaling effects relevant to cognition. Exogenous ketones (EK) offer a practical means of increasing circulating ketone concentrations without dietary carbohydrate restriction. However, the overall effect of EK supplementation on cognitive performance in humans has not been systematically quantified.

**Methods:**

A systematic review and meta-analysis were conducted in accordance with PRISMA guidelines. PubMed, Web of Science, and Embase were searched through October 2025 for randomized controlled trials investigating the effects of EK on cognitive outcomes in healthy adults or individuals with neuropsychiatric conditions. Data extraction and quality assessment were performed independently by multiple reviewers using the PEDro scale. Standardized mean differences (SMD) were calculated using random-effects models. Subgroup and meta-regression analyses examined the influence of ketone formulation, intervention duration, dose, population type, and presence of acute cognitive stressors.

**Results:**

38 studies comprising 41 protocols (1,602 participants) were included in the systematic review, with 29 protocols (1,117 participants) eligible for meta-analysis. EK supplementation was associated with a statistically significant improvement in cognitive performance compared with placebo (SMD = 0.29, 95% CI 0.16–0.41; *p* < 0.001). Sub-group analyses did not show statistically significant differences between the type of supplementation (*p* = 0.083), study duration (acute vs. intermediate; *p* = 0.11), population type (healthy vs. Alzheimer’s disease; *p* = 0.077), or the presence of acute cognitive stressors (*p* = 0.89). Meta-regression revealed a positive association between daily EK dose and cognitive improvement.

**Discussion:**

EK supplementation is associated with modest improvements in cognitive performance across diverse populations and study designs. These findings support EK as a flexible nutritional strategy for cognitive support and warrant further investigation in well-powered, long-term trials to clarify optimal dosing, formulation, and clinical applicability.

**Systematic review registration:**

https://www.crd.york.ac.uk/PROSPERO/view/CRD42023471727, CRD42023471727.

## Introduction

Cognitive function is fundamental to independence, quality of life, and healthy aging, and is influenced by both physiological and pathological processes across the lifespan. Declines in cognitive performance are particularly prominent in neurodegenerative conditions such as Alzheimer’s Disease and related dementias (ADRD), which represent a major public health challenge for aging populations in countries including the USA, UK, and Japan (1). In addition to the reduced quality of life and wellbeing for the patient, ADRD also imposes a cost on society through the significant financial burden of patient care (2). Although pharmaceutical industries have tried to address these challenges by developing both symptomatic and disease-modifying treatments, any such treatments have to be considered in the context of possibly harmful side-effects and practicalities of long-term administration. For these reasons, there has been increasing interest in non-pharmacological strategies that may support cognitive function, with relevance not only to neurodegenerative disease but also to cognitive health more broadly (3).

The canonical primary role of ketone bodies (or ketones) including *β*-hydroxybutyrate (BHB), acetoacetate (AcAc), and acetone, is to act as a metabolic substrate for brain function during development, and in settings of low carbohydrate availability. Indeed, classic experiments by Owen et al. (4) found that ketones entering the brain via select, widely expressed mono-carboxylate transporters (5) can account for up to 60% of brain metabolic needs during prolonged starvation. Alongside their role as an alternative substrate, ketones have been hypothesized to have multiple non-energy, signaling effects in the brain, including increasing cerebral blood flow (6–9), modulating release of neurotransmitters (10, 11) and neurotrophins (12, 13), and altering proteostasis (9, 14, 15). Taken together, there is a strong mechanistic rationale supporting functional benefit of strategies that increase ketone availability to influence cognitive function.

Ketones are produced endogenously, as a result of increased peripheral lipolysis and hepatic conversion of free fatty acids to ketone bodies (16). Ketosis is typically defined as a blood BHB concentration of > 0.5 mM (17–20). Endogenous ketone production increases as a result of dietary strategies that restrict carbohydrate intake, such as during voluntary or involuntary fasting (21) or with consumption of a low-carbohydrate, high-fat, ketogenic diet (17). A healthy adult can produce up to ~150 g of ketone bodies during a prolonged fast (22), reaching a physiological ketosis of 5–7 mM (21). In contrast, long-term consumption of a well formulated ketogenic diet results in a more modest ketosis of < 1 mM (17). While dietary strategies to augment ketosis are increasingly used in both clinical and research context, challenges include long term adherence and concerns regarding the cardiometabolic effects of poorly formulated ketogenic diets.

Exogenous ketones (EK) represent an alternative to existing dietary strategies and can increase circulating ketone concentrations to 0.5–5 mM in a rapid and dose-dependent manner, even when consumed alongside carbohydrate that would usually prevent endogenous ketosis (22–24). Because EK increase ketone concentrations without the concomitant systemic effects of carbohydrate- or calorie- restriction, the metabolic and functional effects of ketogenic diets are distinct from those of EK. There are several types of EK compounds that either directly contain ketone bodies (i.e., free BHB acid, BHB or AcAc mineral salts, esters of BHB or AcAc), or contain precursors that are readily metabolized into ketone bodies (i.e., ketogenic medium chain triglycerides (MCT), medium chain fatty acid esters and ketogenic alcohol (R)-1,3-butanediol). As a category, EK have no currently known safety concerns and, although some gastrointestinal symptoms can occur which may reduce long term compliance, these appear to be reduced by a gradual increase in daily dose (25–27). In the last decade, there has been a steady increase in the number of publications that have addressed the possible physical and cognitive effects of different EK compounds in healthy adults and clinical populations.

As a result of the known metabolic and signaling effects on ketone bodies within the brain, there has been a growing scientific and medical interest in understanding the impact of ketone bodies on cognitive function across both healthy and clinical populations. Given the higher chance of detecting functional improvements when there is an existing deficit, and the prominent role of deficits in brain energy metabolism in neurodegenerative disease (28), many studies to date have understandably focused on disease cohorts. In a key recent systematic review, ketogenic diet and other ketogenic interventions were suggested to positively impact cognition in patients with mild cognitive impairment (MCI) or Alzheimer’s Disease (AD) (29). Importantly, emerging evidence also indicates that ketone availability may influence cognitive performance in non-diseased populations; a recent triangulation of observational studies and mendelian randomization studies found that increased circulating BHB improved general cognitive function as well as delaying the risk of cognitive decline and AD (30). A recent systematic review of studies implementing ketogenic diets found a positive effect on cognition in 80% of included research (31). However, to our knowledge no publications to date have performed a systematic review or meta-analysis to consolidate the evidence for the cognitive effects of EK alone on human cognition, either in individuals with cognitive impairment or in healthy adults.

To address this gap, we undertook this systematic review and metanalysis to evaluate the literature on the effect of EK on human cognition. Given the strong mechanistic evidence for ketones as a fuel and a signal in the brain, we aimed to determine whether EK had a beneficial effect on cognition. If a positive effect was found, we further planned to address if there was an impact of dose or EK compound, duration of intervention, as well as if any benefit was present in healthy individuals, as well as patients with neuropsychiatric disorders.

## Methods

This systematic review was conducted in accordance with the Preferred Reporting Items for Systematic Reviews and Meta-Analyses (PRISMA) checklist (29) and was registered on PROSPERO (registration ID: CRD42023471727). The software program, Rayyan (30), was used during all stages of study selection and data extraction. For the present study, no ethics committee approval was necessary.

### Search strategy

Records were initially searched on three databases (PubMed electronic database of the National Library of Medicine, the Web of Science database, and Embase) to identify eligible studies published before 9th October 2025. MeSH terms and free words referring to ketone bodies (‘ketone bodies’ OR ‘ketosis’ OR ‘ketogenic’ OR ‘*β*-hydroxybutyrate’ OR ‘BHB’ OR ‘beta-hydroxybutyrate’ OR ‘acetoacetate’ OR ‘ketone’ OR ‘exogenous ketone’ OR ‘ketone ester’ OR ‘ketone salt’ OR ‘medium chain triglyceride’ OR ‘MCT’), cognition ((“cognit*” OR “memory” OR “learning” OR “attent*” OR “intellect” OR “executive funct*” OR “recognit*” OR “IQ” OR “problem solving” OR “psychomotor speed” OR “mental flexib*” OR “choice react*” OR “emotional bias” OR “planning” OR “response inhibition”)), and health-related conditions (‘dementia’ OR ‘mild cognitive impairment’ OR ‘MCI’ OR Alzheimer’s Disease’ OR ‘AD’ OR ‘Parkinson’s Disease’ OR ‘PD’ OR ‘traumatic brain injury’ OR ‘TBI’ OR ‘concussion’ OR ‘CTE’ OR ‘stroke’, OR ‘multiple sclerosis’ OR ‘MS’) were used as keywords. References from selected papers and from other relevant articles were screened for potential additional studies in accordance with the snowball principle. Search was limited to journal articles published in English.

Two reviewers (EBS or ACB, and BJS) independently screened titles and abstracts according to the inclusion/exclusion criteria. In the case of conflicts, both reviewers met to discuss their study selection decisions until they reached a mutual decision. Studies that were included based on title and abstract, advanced to full-text screening where the same process was followed (EBS or ACB and BJS review independent, resolved conflicts together). A reason for exclusion was provided during full-text screening. The final analyses were conducted using studies that advanced through both levels of screening.

### Eligibility criteria

A PICOs approach was used as inclusion and exclusion criteria:

- *Participants*: Healthy participants and patients with neuropsychiatric conditions.
- *Intervention*: The intervention being studied was exogenous ketone products. This term encompasses any product that is administered with the aim of increasing blood ketone concentrations. These may include, but not be limited to ketone esters, ketone monoester, ketone di-ester, butanediol, ketone salt, medium chain triglycerides, tri-octanoate, coconut oil, ketone infusion.
- *Control*: Placebo
- *Outcomes*: Outcomes encompassed assessments of one or more cognitive functions conducted both prior to and following ketone body supplementation. These outcomes included a comprehensive evaluation of global cognition, as well as various subdomains of cognitive function, which include verbal memory, nonverbal memory, working memory, processing speed, attention, language proficiency, visuospatial skills, and executive functions. The evaluation of cognitive functions must have been conducted by qualified healthcare professionals such as psychologists, medical doctors, or neuropsychologists
- *Study design*: RCTs

**Figure 1:**
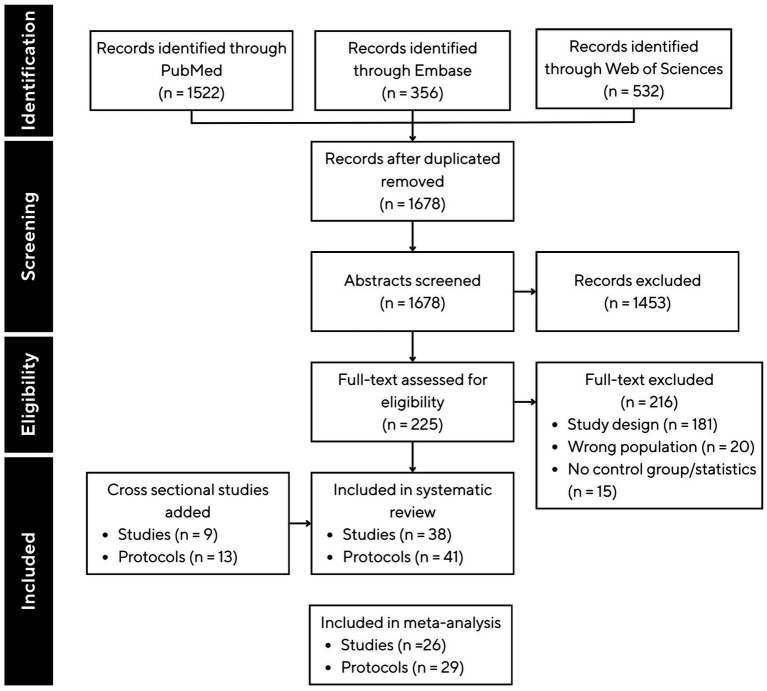
PRISMA flow chart showing study selection process for inclusion in the systematic review and meta-analysis.

A flow diagram of the study selection with the screened articles and the selection process is presented in Figure 1.

### Data extraction

Data were extracted independently by a single reviewer (EBS) and confirmed by a second reviewer (BJS) to verify correct data extraction. The following information was extracted from the included studies: characteristics of the patients (age, sex ratio, general information about conditions/disease), characteristics of the intervention (molecule, dose, duration), type of control and outcomes measurements. Means and error (as standard error, standard deviation, or 95% CI) for cognitive function scores were extracted either by directly extracting from tables/text or by using the data extraction tool, WebPlotDigitizer (WebPlotDigitizer, Pacifica, CA, United States), when data were only reported in figures. Participant population, number of participants, and any co-interventions were also extracted. When a single study evaluated more than one distinct experimental condition or population (e.g., different intervention durations, dosing regimens, or acute versus longer-term protocols), each was treated as a separate protocol for the purposes of subgroup, sensitivity, and meta-regression analyses, while participant counts reflected unique individuals and were not duplicated across protocols.

### Quality assessment

The PEDro scale, which is deemed a valid and reliable tool for assessing RCTs, was used for methodological quality assessment. RCTs’ quality was blindly judged by two different reviewers (BJS and EBS) to minimize potential bias. To ensure the rigor of this process, the final decision about each RCT quality was made by reaching a consensus. In case of discordance, a third collaborator (BB) was consulted to provide expert input. The RCTs were classified into distinct categories based on their quality: low quality (scores falling within the range of 0 to 3 out of 10); moderate quality (scores spanning 4 to 6 out of 10) and high quality (RCTs achieving scores from 7 to 10 out of 10). This methodical approach allowed for a comprehensive evaluation of the RCTs, thus facilitating an objective assessment of their respective quality levels.

### Statistical analysis

For studies assessing the efficacy of ketone bodies and displaying complete results of the pre and post-tests, we performed a meta-analysis. The measure of treatment effect was the standardized mean difference effect size (standardized mean difference [SMD]), defined as the between-group difference in mean values divided by the pooled SD computed using the Hedge’s g method. If different tests were used to assess the cognitive in the same study, the different results were pooled to have one unique SMD as recommended by Cochrane’s group. A positive SMD implies an increased cognitive function in comparison with the control group. Subgroup analyses were conducted to explore potential sources of heterogeneity, including intervention duration, ketone formulation, participant health status, and presence of acute cognitive stressors; only subgroup categories represented by more than two independent studies were included in these analyses. We calculated the variance estimate tau^2^ as a measure of between-trial heterogeneity. We prespecified a tau^2^ of 0.0 to represent no heterogeneity, 0.0–0.2 to represent low heterogeneity, 0.2–0.4 to represent moderate heterogeneity, and above 0.4 to represent high heterogeneity between trials. To deal with high or moderate heterogeneity we used random-effect models and presented forest plots for the different comparators. We checked for publication bias using funnel plot (15) and Egger’s test for the intercept was applied to check the asymmetry (16) and performed sensitivity analysis to detect potential outliers. Meta-regression was performed to assess a possible effect of the dose, the duration (long term = > 13 days; immediate = single administration and same-day measurement) of the intervention and the type of supplementation on the results. While blood ketone concentration would be the preferred variable for correlation with functional outcomes, given the known differences in ketogenic effect of different types of EK (22, 32), ketone concentrations were not measured in all studies, and when included they were not measured at a standardized time. Given that blood ketone concentrations can change dramatically over 30 min, dose consumed was chosen for analysis.

The statistics were conducted in RStudio (version 2026.01.0) with R version 4.5.2 and the significance level set at *p* < 0.05.

## Results

### Search results

Across 38 eligible studies, a total of 41 distinct experimental protocols were identified, as several studies included multiple intervention arms or testing durations; participant counts reflect unique individuals and were not duplicated across protocols. The PRISMA flowchart of the study selection is presented in Figure 1.

#### Characteristics of the participants

Table 1 provides a comprehensive overview of the studies included in the analysis, detailing their respective characteristics. In total 1,602 participants were included in this review, among them 1,538 (96%) completed the full protocol. The complete characteristics of the participants and interventions are presented in Table 1.

**Table 1:** Characteristics of studies included in the systematic review and meta-analysis.

Study	Country	Participants, n (female)	Age (y)	Characteristics	Molecule	Dose	Duration	Control	PEDRO
Abe et al., 2020 (33)	Japan	64, (51)	85.5 ± 6.8	BMI 18.6 ± 2.5 Participants resided in nursing home. Required special care from helper Excluded BMI > 23 Excluded age <65 Excluded fasting blood glucose (> = 200 mg/dL), blood creatinine (> = 1.5 mg/dL), and C-reactive protein (> = 2.0 mg/dL)	MCT (75% 8:0, 25% 10:0)	6 g/d	3 months	Positive control: MCT (6 g) + L-leucine (1.2 g) + cholecalciferol (20ug) Negative control: LCT (6 g, [64% 18:1, 19%18:2, 9% 18:3])	6
Ashton et al., 2020 (50)	UK	30, (14)	19.7 ± 1.5	Healthy university students	MCT (30% 8:0, 70% 10:0)	12 g/d; 18 g/d	4 weeks	Isocaloric carbohydrate gel	6
Baranowski (14-day protocol) (40)	Canada	15, (6)	56.6 ± 9.1	30–70 yro T2D based on A1C > =6.5% or fasting plasma glucose > = 7.0 mmol/L excluded athletes, exogenous insulin and SGLT2 inhibitors, keto/lowcal/fasting/ketogenic supplement diets	(R)-3-hydroxybutyl (R)-3-hydroxybutyrate ketone monoester (KetE)	45 g/d	14 days	Placebo	7
Baranowski (acute protocol) (40)	Canada	18, (7)	62 ± 8	30–70 yro T2D based on A1C > =6.5% or fasting plasma glucose > = 7.0 mmol/L excluded athletes, exogenous insulin and SGLT2 inhibitors, keto/lowcal/fasting/ketogenic supplement diets	(R)-3-hydroxybutyl (R)-3-hydroxybutyrate ketone monoester (KetE)	0.3 g*kg-1 body mass	1 time	Placebo	9
Choi (14-day protocol) (51)	USA	16, (7)	67.1 ± 4.1	>50 yro clinically probably diagnosis of PD able to walk independently excluded other neurodegenerative disease, kidney disease, liver disease, uncontrolled diabetes, hyperlipidemia	Ketogenic diet+MCT	30 mL MCT oil per meal, 20 mL per snack	14 days	Placebo	7
Choi (7-day protocol) (51)	USA	16, (7)	67.1 ± 4.11	>50 yro clinically probably diagnosis of PD able to walk independently excluded other neurodegenerative disease, kidney disease, liver disease, uncontrolled diabetes, hyperlipidemia	Ketogenic diet+MCT	80% energy from fat, 25% of fat from MCT	7 days	Standard diet	7
De la Rubia Orti et al., 2018 (35)	Spain	44, (3)	84	Diagnosed with AD excluded other degenerative cognitive disorders excluded verbal disability excluded treatment with drugs that could alter cognitive function	Coconut oil	36 g/d	21 days	Mediterranean diet	6
Evans et al., 2018 (41)	Ireland	11, (0)	25.4 ± 4.6	Team sport athletes height 1.80 ± 0.05 m body mass 78.6 ± 5.3 kg VO2max 53.9 ± 2.2 actively training and competing in high intensity field-based team sports	R-BHB (R)1,3-butanediol ketone ester (KetE)	750 mg*kg-1	Before and during exercise	6.4% carbohydrate electrolyte solution	8
Evans et al., 2019 (43)	Ireland	8, (13)	33.5 ± 7.3	Trained middle- and long-distance runners height 1.79 ± 0.07 m body mass 68.8 ± 9.7 kg body fat 8% ± 4.1% VO2max 62.0 ± 5.6	(R)-3-hydroxybutyl (R)-3-hydroxybutyrate ketone monoester (KetE)	573 mg*kg-1	Before and during exercise	8 carbohydrate electrolyte solution	9
Fortier et al., 2019 (25)	Canada	52, (21)	75.4 ± 6.6	MCI MoCA 18–26 MMSE 24–27 GDS < 10 Autonomy of ADL	Ketogenic MCT (kMCT), 12% Captex 355 (60% caprylic acid, 40% capric acid)	30 g/d	6 months	High-oleic acid sunflower oil	8
Fortier et al., 2021 (34)	Canada	82, (45)	71.4 ± 7.2	MCI based on Peterson criteria MoCA 18–26 MMSE 24–27	Ketogenic MCT (kMCT), 12% Captex 355 (60% C8, 40% C10)	30 g/d	6 months	High-oleic acid sunflower oil	8
Graybeal et al., 2025 Metabolic syndrome participants) (38)(	USA	10, (3)	29 ± 12	18–55 yro With MetS	(R)-3-hydroxybutyl (R)-3-hydroxybutyrate ketone monoester (KetE)	0.282 g*kg-1 body mass	1 time	Placebo	6
Graybeal et al., 2025 (non-metabolic syndrome participants) (38)	USA	10, (3)	29 ± 12	18–55 yro Without MetS	(R)-3-hydroxybutyl (R)-3-hydroxybutyrate ketone monoester (KetE)	0.282 g*kg-1 body mass	1 time	Placebo	6
Heidt et al., 2023 (32)	Germany	19, (12)	24.4 ± 3.9	Healthy	MCT (60% 8:0, 40% 10:0)	0.5 g/kg; 0.5 g/kg + 0.2 g/kg glucose	1 time	200 mL still drinking water	6
Henderson et al., 2009 (36)	USA	152, (85)	76.9 ± 8.9	Mild to moderate AD MMSE score 14–24 excluded depression, hypothyroidism, B12 deficiency, renal disease or insufficiency, hepatic disease or insufficiency, and diabetes.	AC-1202 (1,2,3-propanetriol Tri octanoate, 8:0)	20 g/d	90 days	Powder: 51% gum acacia, 37% dextrose, 10% safflower oil, 2% syloid	9
Henderson et al., 2020 (52)	USA	413, (245)	76.3 ± 6.44	Included AD excluded any other neurological condition excluded depression, stroke	AC-1204 (caprylic triglyceride)	20 g/d	6 months	Sunflower oil and maltodextrin	8
Lee et al., 2021 (53)	USA	15, (7)	51.9 ± 10.1	Primary progressive MS or SPMS 30-65yro walk 25 ft. in <60s severe fatigue	MCT-based ketogenic diet	54–67.5 g/d	12 weeks	Regular diet	6
McClure et al., 2024 (54)	USA	23, (0)	25.3 ± 2.3	18–35 yro standard American diet excluded smoking, metabolic or cardiovascular disease, exercise limiting conditions	(R)-3-hydroxybutyl (R)-3-hydroxybutyrate ketone monoester (KetE)	0.650 g*kg-1 body mass	1 time	Placebo	6
McClure et al., 2024 (40)	USA	16, (0)	24.4 ± 4.3	18–35yro standard American diet excluded smoking, metabolic or cardiovascular disease, exercise limiting conditions	(R)-3-hydroxybutyl (R)-3-hydroxybutyrate ketone monoester (KetE)	0.573 g*kg-1 body mass	1 time	Placebo	7
Mutoh et al., 2022 (55)	Japan	63, (22)	69.8 ± 4.0	65-80yro right-handed global dementia rating = 0 MMSE> = 24	MCT	18 g/d	3 months	Canola oil	10
Nishioka et al., 2025 (56)	Japan	40, (24)	52.1 ± 6.4	40-60yro excluded: restrictive diet, ketone or MCT supplementation	3-hydroxybutyrate	3.5 g	1 time	Placebo	10
Ohnuma et al., 2016 (57)	Japan	22, (10)	63.9 ± 8.5	mild–moderate AD MMSE 10–26 no family history of AD	Caprylic triglycerides	20 g/d	90 days	None	4
Ota et al., 2019 (58)	Japan	20, (9)	73.4 ± 6	Mild–moderate AD	MCT	20 g/d	12 weeks	Placebo	7
O’Neil et al., 2019 (59)	UK	96, (40)	65.4 ± 6.19	Healthy 55-80yro MMSE > = 25 no neurological or psychiatric disorder	MCT (55% 8:0, 45% 10:0)	30 g/d	14 days	Placebo	10
Page et al., 2009 (46)	USA	11, (6)	34.8 ± 8.9	Intensively treated type 1 diabetics	MCT (67% 8:0, 27% 10:0, 6% other fatty acids)	40 g	180 min	Crossover/euglycemia	9
Poffe et al., 2023 (60)	Belgium	18, (0)	37.1 ± 7.2	Recreational runners VO2max 53.6 +/− 2.8	R-BHB R-1,3 butanediol (KetE)	430 g total	1 time (over 2 days)	Control drink	8
Prins et al., 2020 (61)	USA	13, (0)	24.8 ± 9.6	Distance runners 5 k under 25 min	BHB-salt + MCT	7 g BHB + 7 g MCT; 14 g BHB + 7 g MCT	1 time	Water with MiO	9
Prins et al., 2021 (44)	USA	15, (0)	20.6 ± 2.1	Recreational distance runners	(R)-3-hydroxybutyl (R)-3-hydroxybutyrate ketone monoester (KetE)	573 mg*kg-1	1 time	Placebo	9
Quinones et al., 2022 (62)	Canada	9, (0)	30 ± 3	Recreationally active soccer players	Ketone monoester (KetE)	25 g	1 time	Placebo	9
Rebello et al., 2015 (63)	USA	6, (2)	58–78	MCI > =50 yro	MCT	56 g/d	24 weeks	Canola oil	5
Reger et al., 2004 (64)	USA	20, (10)	74.7 ± 6.7	MCI or AD	MCT	37.8 g	1 time	Heavy whipping cream	9
Roy et al., 2022 (65)	Canada	32, (8)	74.2 ± 6.3	MCI > =55 yro	Ketogenic (MCT) kMCT	30 g/d	6 months	Sunflower oil	8
Stalmans et al., (66)	Belgium	14, (0)	26 ± 5	18–35yro BMI 18–25	(R)-3-hydroxybutyl (R)-3-hydroxybutyrate ketone monoester (KetE)	75 g	1 time	Placebo	9
Veneman et al., 1994 (67)	US	13, (5)	33 ± 3	BMI 28 ± 1	DL-BOHB (ketone salt)	20umol*kg*min	360 min	Na-L-lactate	7
Waldman et al., 2018 (68)	USA	15, (0)	23.1 ± 2.4	Healthy	BHB Perfect Keto Bolt Themes (ketone salt)	11.38 g	1 time	Placebo	9
Waldman et al., 2019 (69)	USA	16, (0)	21.9 ± 1.9	Healthy, recreationally active	BHB Ketone Salt	0.38 g*kg-2 body mass	1 time	Placebo	9
Waldman et al., 2024 (70)	USA	12, (12)	23 ± 3	Division 1 athletes or actively training in running or cycling	(R)-3-hydroxybutyl (R)-3-hydroxybutyrate ketone monoester (KetE)	0.375 g*kg-1 body mass	1 time	Placebo	8
Xu et al., 2020 (37)	China	53, (31)	75.1 ± 7.5	Mild–moderate AD MMSE 14–24	MCT	17.3 g/d	30 days	Canola oil placebo	10
Yomogida et al., 2021 (71)	Japan	20, (14)	65.7 ± 3.9	No psychiatric disorders MMSE > = 26	MCT	19.9 g	1 time	Placebo meal, LTCs only	10
Yu et al., 2025 (Healthy Weight participants) (39)	China	40, (20)	23.8 ± 3.9	right-handed 18–35yro BMI > 18 & < 25	R-1,3-butanediol	0.395 g*kg-1 body mass	1 time	Placebo	8
Yu et al., 2025 (Overweight participants) (39)	China	40, (20)	23.8 ± 3.9	right-handed 18–35yro BMI > 25	R-1,3-butanediol	0.395 g*kg-1 body mass	1 time	Placebo	8

**Figure 2:**
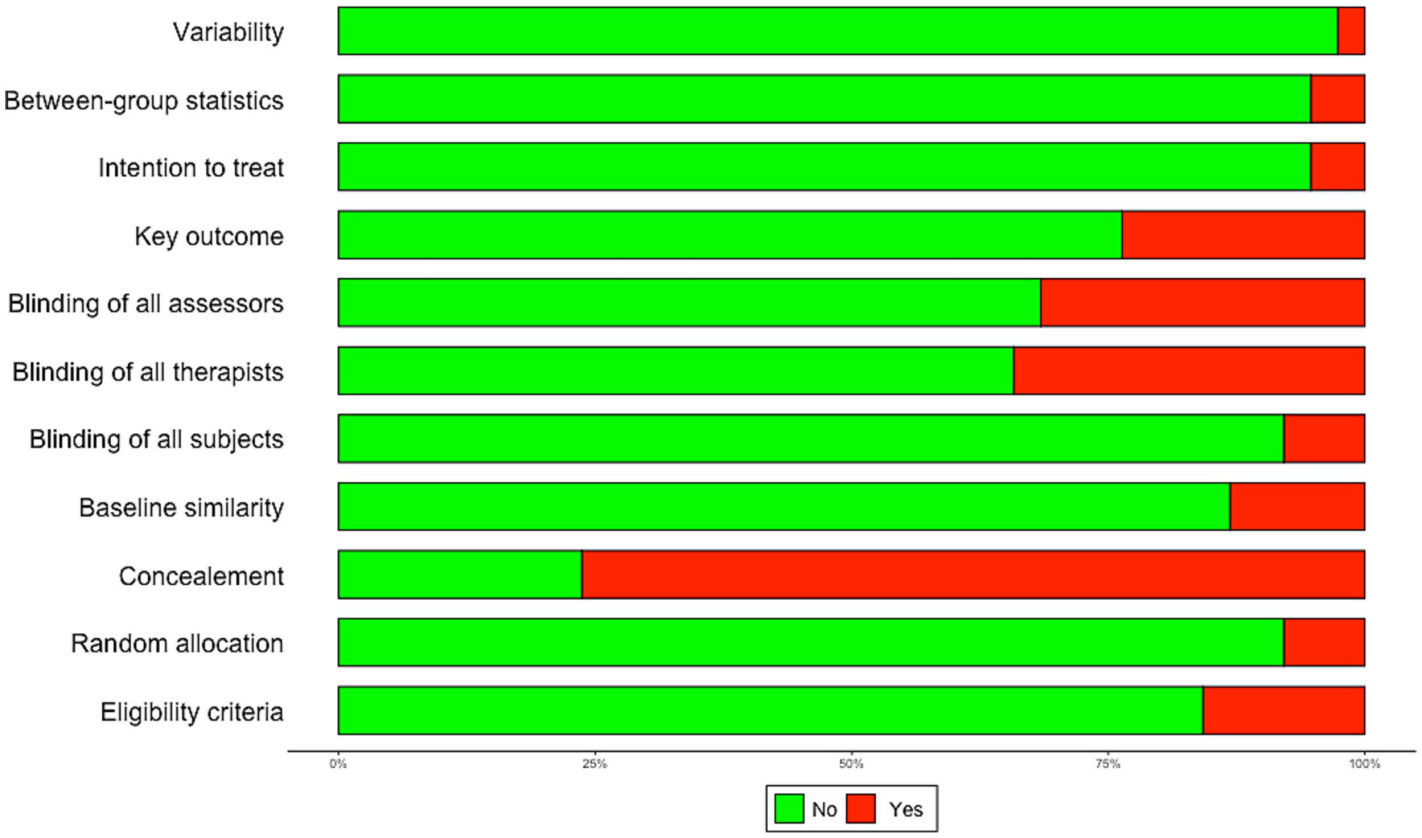
PEDRO scores for quality domains.

Concerning the quality of the individual studies we found an overall PEDRO score of 7.9 (1.6) out of 10 indicating high quality studies, with only 7 studies being judged as moderate quality. Individual results are presented in Table 1 and summarized in Figure 2.

### Systematic review

#### Characteristics of the intervention

The studies reviewed encompass a wide range of geographic locations, with the largest number of participants coming from the United States, which contributed 792 participants across 17 studies. China followed with 373 participants in only 3 studies, Japan followed with 229 participants across 6 studies, while Canada included 223 participants in 7 studies. Other countries with fewer participants included the United Kingdom (126 participants across 2 studies), Belgium (32 participants in 2 studies), Ireland (19 participants across 2 studies), Spain (44 participants in 1 study), Germany (19 participants in 1 study).

The types of supplementations presented high heterogeneity, with lipid-based precursors being the most common. MCTs were used in 1,054 participants across 19 studies, with dosages that ranged from 6 g/day to 56 g/day, with common formulations including caprylic acid (C8:0) and capric acid (C10:0). Coconut oil was used in a single study with 44 participants, administered at a dosage of 36 g/day, in the context of AD. AC-1202 and AC-1204 (proprietary, purified caprylic triglycerides), were used in studies involving 565 participants across 2 studies in the United States. Non-lipid-based ketone supplements included BHB salts, which were used in 44 participants across 2 studies, primarily in athletic performance contexts, and a ketone ester (KetE; (*R*)-3-Hydroxybutyl-(R)-3-Hydroxybutyrate), involving 300 participants across 15 studies. The dosages for the EK interventions varied, typically ranging from 573 mg/kg to 162.5 g total, and were predominantly used in studies focusing on athletic performance and cognitive function under stress.

The duration of the interventions also varied widely. The intervention duration ranged from single-dose studies to longer studies lasting up to 6 months, with the most common durations being between 1.5 to 3 months.

The most common outcomes evaluated across these studies focused on cognitive function and physical performance. Cognitive function was assessed using various tools, with the Mini-Mental State Examination (MMSE) and Alzheimer’s Disease Assessment Scale-Cognitive Subscale (ADAS-Cog) being the most frequently used. Neuropsychological tests such as the Trail Making Test and Stroop Test were also commonly employed. These assessments were applied across diverse populations, including healthy adults, individuals with MCI, and patients with AD. Physical performance outcomes were primarily measured in athletes and healthy adults, focusing on endurance performance (e.g., time trials), muscle strength (e.g., grip strength, knee extension time), and reaction times. Additionally, biomarkers like plasma BHB levels were often measured to confirm ketosis, alongside glucose and lactate concentrations, particularly in studies involving KetE.

#### Clinical efficacy

Our interest focused on the clinical effects of ketone supplementation on cognitive performance. The relevant outcomes and main results of the included studies are presented in Table 2.

**Table 2:** Description of participants’ outcomes and main tests of the included studies.

Study (Protocol)	Summary (duration, type, Participant health status, stressor)	Outcome and tests	Main results	Conclusion
Abe et al., 2020 (33)	Long-term, MCT, healthy, no stressor	Primary Tests: 10-s leg open/close test, MMSE Secondary Tests: Upper-arm circumference, bilateral calf circumference, triceps skinfold thickness, mid-upper-arm muscle area, hand-grip strength, knee extension time, walking speed, peak expiratory flow, RSST (swallowing function), FIM (ADL)	MCT supplementation increased total MMSE score by 3.5 points from BL to 3-mo intervention	Supplementation with 6 g MCT may improve cognition in frail older adults
Ashton et al., 2020 (50)	Long-term, MCT, healthy, no stressor	Primary Tests: Trail Making Test, Digit Span Test (forward/backward), Spatial Span Test, Covert Shift of Attention, Rapid Visual Information Processing	After 2–3 weeks of MCT supplementation, performance improved in cognitive tasks (trail making a/b, digit span forwards/backwards). There were minimal differences between the different doses	MCT ingestion improved cognitive performance after 2–3 weeks, minimal difference between 12 g/d and 18 g/d
Baranowski (14-day protocol) (40)	Immediate, KetE, T2DM, no stressor	Primary Tests: DSST, Stroop Test, Task-switching	B-OHB did not alter recognition memory	BOHB supplementation did not improve cognition or elevate BDNF content
Baranowski (acute protocol) (40)	Long-term, KetE, T2DM, no stressor	Primary Tests: N-back memory task, DSST	B-OHB did not alter recognition memory	BOHB supplementation did not improve cognition or elevate BDNF content
Choi (14-day protocol) (51)	Long-term, ketogenic diet + MCT, PD, no stressor	Primary Tests: Feasibility and acceptability Secondary Tests: Timed up and go Exploratory Tests: N-back, Unified Parkinson’s Disease Rating Scale, Non-motor Symptom Scale, rsEEG connectivity	ketosis achieved by day 4, TUG no change, 3-back no change	ketogenic diet+MCT is feasible and acceptable, further studies to understand functions in PD
Choi (7-day protocol) (51)	Long-term, ketogenic diet + MCT, PD, no stressor	Primary Tests: feasibility and acceptability Secondary Tests: change in Timed up and go Exploratory Tests: N-back, unified Parkinson’s Disease rating scale, non-motor symptom scale, rsEEG connectivity	TUG no change, nonmotor symptom severity score reduced in KD	ketogenic diet+MCT is feasible and acceptable, further studies to understand functions in PD
De la Rubia Orti et al., 2018 (35)	Long-term, coconut oil, AD, no stressor	Primary Tests: Benton’s Temporal Orientation Test, Clock Drawing, Categorical Verbal Fluency, Free and Cued Selective Reminding Test	After intervention, episodic, temporal orientation, and semantic memory improved. Effect is more evident in women with mild–moderate state AD	The coconut oil enriched mediterranean diet improves cognitive function in patients with AD
Evans et al., 2018 (41)	Immediate, KetE, healthy, exercise	Primary Tests: Reaction Time Test Secondary Tests: VO2max, Loughborough Intermittent Shuttle Test (LIST)	The KetE resulted in increased plasma BHB concentrations. Plasma glucose and lactate concentrations were lower in the KetE group. HR, RPE, and 15 m sprint times did not differ. Run time to exhaustion was not different. Incorrect responses in a multitasking test increased in the PLA group but not KE	KetE ingestion attenuated a decline in executive function after exhausting exercise, suggesting a cognitive benefit
Evans et al., 2019 (43)	Immediate, KetE, healthy, exercise	Primary Tests: 10 km time trial Secondary Tests: Cognitive performance tests, oxygen consumption, running economy, respiratory exchange ratio (RER), HR, RPE, plasma BHB, glucose, lactate concentrations	Following KetE ingestion, plasma BHB concentrations increased, but glucose and lactate concentrations were similar. No other measures differed	KetE ingestion by endurance-trained athletes elevated plasma BHB concentrations but did not improve 10 km running time trials or cognitive performance
Fortier et al., 2019 (25)	Long-term, MCT, AD, no stressor	Primary Tests: Positron Emission Tomography (PET) for brain metabolism Secondary Tests: RL/RI16, Brief Visual Memory Test-Revised, Trail Making Test, Stroop Color and Word Interference, Verbal Fluency, Digit Symbol Substitution, Boston Naming Test	Brain ketone metabolism increased by 230% for kMCT. kMCT improved measures of episodic memory, language, executive function, and processing speed	This dose and duration improve several cognitive outcomes in MCI
Fortier et al., 2021 (34)	Long-term, MCT, AD, no stressor	Primary Tests: RL/RI16, Verbal Fluency, Boston Naming Test, Trail Making Test	Free and cued recall, verbal fluency, Boston naming test, and trail making tests improved with kMCT. Some cognitive outcomes correlated positively with plasma ketones	This drink improved cognitive outcomes in MCI
Graybeal et al., 2025 healthy and metabolic syndrome participants) (38)	Immediate, KetE, cognitively healthy, no stressor	Primary Tests: Stroop Test, Task-switching, Go no-go, Digit span test, 1 and 2 N-back Test	Following KetE ingestion, working memory improved and blood glucose decreased. Appetite only decreased in non-MetS group	Cognition may improve following KetE ingestion regardless of MetS status, but appetite only improves in those without MetS
Heidt et al., 2023 (32)	Immediate, MCT, healthy, no stressor	Primary Tests: Wechsler Adult Intelligence Scale-IV (WAIS-IV) Verbal Comprehension Index (VCI), Working Memory Index (WMI) Secondary Tests: Satiety and tolerability questionnaires, plasma BHB levels	Plasma BHB increased after ingestion of MCT oil, and a delayed increase with MCT + glucose. MCT oil plus glucose showed improved scores for the cognitive tests	For MCT + glucose consumption, there were fewer side effects and positive effects on cognitive ability, and similar trends were also observed for MCT alone
Henderson et al., 2009 (36)	Long-term, MCT, AD, no stressor	Primary Tests: ADAS-Cog, ADCS-CGIC Secondary Tests: Serum BHB levels	Molecule elevated serum BHB. AC-1202 group had significantly different mean change in ADAS-Cog	AC-1202 rapidly elevated serum ketone bodies in AD patients and resulted in significant differences in ADAS-Cog scores compared to the Placebo. Effects were most notable in APOE4(−) subjects who were dosage compliant
Henderson et al., 2020 (52)	Long-term, MCT, AD, no stressor	Primary Tests: ADAS-Cog11 Secondary Tests: ADCS-ADL, Clock Drawing, Resource Utilization in Dementia (RUD-Lite), QoL in Alzheimer’s Disease (QoL-AD), MMSE	AC-1204 was tolerated. Mean changes in ADAS-Cog11 for placebo was 0.0, AC-1204 was 0.6	AC-1204 did not improve cognition or functional abilities in subjects with mild–moderate AD
Lee et al., 2021 (53)	Long-term, MCT, healthy, no stressor	Primary Tests: Modified Fatigue Impact Scale (FIS), Expanded Disability Status Scale (EDSS) Secondary Tests: MSQoL, Multiple Sclerosis Functional Composite (MSFC)	BHB indicated nutritional ketosis in keto group, in paleo and control, no change. Paleo group had reduction in fatigue scores and maintained cognitive function scores. Keto group had reduction in fasting glucose in insulin	Consuming the MCT-based ketogenic diet achieved nutritional ketosis, but it was not associated with clinical improvement, whereas the paleo diet saw clinical improvements
McClure et al., 2024a (54)	Immediate, KetE, healthy, hypoxia	Primary Tests: RightEye oculometric, Defense Automated Neurobehavioral Assessment (DANA)	Following KetE ingestion, lesser decline in cognitive performance in hypoxia, as well as SpO2 decline	KetE attenuated decline in cognitive performance in acute hypoxic exposure, coinciding with attenuations in declines of O2 saturation
McClure et al., 2024b (40)	Immediate, KetE, healthy, exercise and hypoxia	Primary Tests: DANA, Stroop color and word task, shooting simulation	Following KetE ingestion, outcomes in cognitive performance tasks did not differ	KetE attenuated declines in SpO2 but had no effect on cognitive performance during exercise
Mutoh et al., 2022 (55)	Long-term, MCT, healthy, no stressor	Primary Tests: Brain PET scan, Resting-state fMRI, MMSE Secondary Tests: Wechsler Memory Scale-Revised (WMS-R) Logical Memory (LM) test, Trail Making Test, Digit Span, Digit Symbol Substitution Test (DSST), physical function, and anthropometric measures	MCT group had better balance ability, no cognitive or other gait parameter change. MCT suppressed glucose metabolism	3-month MCT supplementation improves walking balance
Nishioka et al., 2025 (56)	Immediate, 3-HB, healthy, no stressor	Primary Tests: serial arithmetic test, cognitrax	SAT response improved following 3-HB ingestion	3-HB may improve cognitive function and mood in healthy subjects after a single dose of 3-HB
Ohnuma et al., 2016 (57)	Long-term, MCT, healthy, no stressor	Primary Tests: Cambridge Neuropsychological Test Automated Battery (CANTAB), Source Memory Task	Increased plasma BHB concentrations No significant improvements in cognitive function or memory related neuronal activity observed	Increasing plasma BHB levels with this intervention had no effects on cognitive function.
Ota et al., 2019 (58)	Long-term, MCT, AD, no stressor	Primary Tests: Alzheimer’s Disease Assessment Scale-Cognitive Subscale (ADAS-Cog), MMSE	Intervention did not improve cognitive function. Some ApoE4-negative patients with baseline MMSE > = 14 showed improvements	Might be effective for AD patients with MMSE> = 14
O’Neil et al., 2019 (59)	Immediate, MCT, AD, no stressor	Primary Tests: Wechsler Memory Scale-Revised (WMS-R), WAIS-III, Trail Making Test, Stroop Test	No significant difference between the one-time MCT dose and placebo for cognitive function. In 12 week, trial, improvement in Digit symbol coding and immediate logical memory	The chronic consumption of this formula may have positive effects on verbal memory and processing speed in patients with AD
Page et al., 2009 (46)	Immediate, MCT, healthy, hypoglycemia	Primary Tests: Immediate Verbal Memory, Delayed Verbal Memory, Verbal Memory Recognition, Digit Span Backward, Letter/Number Sequencing, Digit Symbol Coding, Map Search (1 min and 2 min), Telephone Search	Hypoglycemia impaired cognitive function. MCT ingestion reversed these effects. MCT increased BHB levels	MCT ingestion improves cognition in response to hypoglycemia in T1D
Poffe et al., 2023 (60)	Immediate, KetE, healthy, exercise	Run until exhaustion, or until 100 km ran Primary Tests: Reaction Time Test, Rapid Visual Information Processing, Spatial Working Memory, Cambridge Gambling Task	In control, RUN increased visual reaction time and movement execution time, however KetE negated this effect	KetE enhances mental alertness in ultra-endurance exercise
Prins et al., 2020 (61)	Immediate, BHB salt + MCT, healthy, exercise	Primary Tests: 5 k time trial Secondary Tests: Automated Neuropsychological Assessment Metric (ANAM)	Increased BHB, exercise performance was unaltered, however there were responders and non-responders. Larger dose augmented cognitive function in pre-exercise conditions, and exercise increased cognitive performance for smaller dose and placebo	This formulation had a dosing effect on cognitive performance, but did not influence exercise performance
Prins et al., 2021 (44)	Immediate, KetE, healthy, exercise	Voluntary hypoventilation with exercise Primary Tests: Automated Neuropsychological Assessment Metric (ANAM)	KetE increased BHB, reduced blood glucose, no changes in lactate production, alterations to pH, bicarbonate, total carbon dioxide, increased respiratory exchange ratio, and blood carbon dioxide was significantly lower immediately post voluntary hypoventilation + exercise. The protocol decreased cognitive performance similarly in both conditions	No difference between KetE and PLA in cognitive decline resulting from voluntary hypoventilation plus exercise.
Quinones et al., 2022 (62)	Immediate, KetE, healthy, exercise	Primary Tests: Stroop Test, Choice Reaction Task	KetE had a reduced performance decrease compared to placebo. No other cognitive function differences see	KetE supplementation attenuated decreases in the choice reasoning task during repeated, high intensity, intermittent exercise
Rebello et al., 2015 (63)	Long-term, MCT, MCI/AD, no stressor	Primary Tests: ADAS-Cog	MCT ingestion increased serum ketones and improved memory	Consumption of 56 g/d of MCT for 24 weeks increases serum ketone concentration and may modulate cognitive function
Reger et al., 2004 (64)	Immediate, MCT, MCI/AD, no stressor	Primary Tests: ADAS-Cog, MMSE, Stroop Test, Paragraph Recall	MCT treatment facilitated higher performance on ADAS-Cog for E4- subjects	Pts with APOE4- patients may benefit from MCTs
Roy et al., 2022 (65)	Long-term, MCT, MCI, no stressor	Primary Tests: Trail Making Test (TMT), Stroop Test, DSST, MRI, PET scan	kMCT was associated with increased functional connectivity within the dorsal attention network, correlating to improvement in cognitive tests targeting attention	Ketones in MCI may be beneficial for cognition
Stalmans et al., (66)	Immediate, KetE, healthy, hypoxia	Primary Tests: RTI, RVP, SWM	EPO increased after 5 h during exercise bouts after ingestion of KetE or placebo	KetE ingestion impairs exercise performance and KetE did not protect against hypoxia induced cognitive decline
Veneman et al., 1994 (67)	Immediate, ketone salt, healthy, no stressor	Primary Tests: Cognitive Deterioration Test	Infusion of BOHB increased the glycemic threshold and reduced magnitude of autonomic and neuroglycopenic symptoms and cognitive dysfunction	BOHB may substitute for glucose as a fuel for the brain and alter physiological responses in hypoglycemia
Waldman et al., 2018 (68)	Immediate, ketone salt, healthy, exercise	Primary Tests: Cognitive Challenge (FitLights), Wingate Trials	No significant differences among Wingate power output between treatments	BHB did not improve high intensity cycling or cognitive performance measures
Waldman et al., 2019 (69)	Immediate, ketone salt, healthy, exercise	Primary Tests: Dual-Stress Challenge, Mental Arithmetic Challenge, Stroop Color-Word Test Secondary Tests: Heart rate, Rating of Perceived Exertion (RPE), Blood BHB levels	Blood BHB was elevated and remained throughout. Blood glucose was lower during KS compared to PLA. No differences in HR, RPE, MAC, or SCW	KS are not effective aids for enhancing cognitive performance during a DSC
Waldman et al., 2024 (70)	Immediate, KetE, healthy, exercise	Primary Tests: cycling performance Secondary Tests: PVT, Task switching, Incongruent flanker	KetE +CHO increased psychomotor vigilance and reaction time, speed, and correct responses	KetE +CHO improved markers of cognitive performance after exercise
Xu et al., 2020 (37)	Long-term, MCT, AD, no stressor	Primary Tests: ADAS-Cog-C Secondary Tests: Activities of Daily Living (ADL) scale	ADAC-Cog-C scores were reduced for MCT intervention. ADL scores did not change	MCT had positive effects on cognition in mild to moderate AD patients
Yomogida et al., 2021 (71)	Immediate, MCT, healthy, no stressor	Primary Tests: Functional MRI (fMRI), Executive Function Tasks (N-back, Go/No-Go)	MCT meal improved N-back task	MCT was associated with heightened cognitive functions in healthy older adults. Potential beneficial impact of ketones with respect to improved cognitive outcomes
Yu et al., 2025 (Healthy Weight participants) (39)	Immediate, KetE, healthy, no stressor	Primary Tests: Functional near-infrared spectroscopy, Stroop Test	KetE ingestion increased causal density and decreased cognitive interference	KetE ingestion may beneficially modulate neurocognition in healthy, normal weight younger adults
Yu et al., 2025 (Overweight participants) (39)	Immediate, KetE, obese, no stressor	Primary Tests: Functional near-infrared spectroscopy, Stroop Test	KetE ingestion increased causal density and decreased cognitive interference	KetE ingestion may beneficially modulate neurocognition in healthy younger adults with obesity

Many of the studies focused on long-term use of MCTs and structurally related EK compounds in older adults Several studies demonstrated improvements in cognitive performance in older adults with frailty, MCI or AD after long-term MCT supplementation (25, 33, 34), or coconut oil enriched Mediterranean diet (35). EK were associated with increased MMSE scores and improvements in episodic memory, language, and executive function. Of note, two studies found that the cognitive benefits of long-term EK might be greater in individuals without the APOE4 allele (36, 37). Only one long-term study utilized a non-MCT product, finding that 14 days of KetE supplementation improved performance of the Digit Symbol Substitution Task in obese adults (8).

Of the studies investigating the immediate effects of EK supplements, a large proportion of these were in a younger, population with metabolic, not neuropsychiatric disease, and utilized KetE. Two studies examined the effects of KetE on cognition in both healthy and overweight or metabolic syndrome participants (38, 39) and found that KetE improved cognition in both groups, however a similar study of KetE in Type 2 Diabetes found no effect with acute or 14 day dosing (40). The results on KetE on cognitive performance during a physiologic stressor (i.e., exercise, hypoventilation or hypoxia) were mixed, with some studies finding KetE-linked attenuation of exercise-induced decline in cognitive function (41) and visual reaction time (38) or hypoxia induced decline in cognition and brain SpO_2_ (42). However, other studies used similar young, healthy populations in the context of a stressor found no protective effect of KetE against cognitive decline (43, 44). Two studies reported that a single MCT dose improved immediate cognitive task performance in AD (45) and in the context of hypoglycemia (46), although the effects were dose-dependent and varied by cognitive domain.

### Meta-analysis

Out of the 41 protocols (from 38 studies) included in the systematic review, 29 protocols were included in the meta-analysis (from 26 studies, some of which included multiple protocols), representing 1,117 participants, to quantify the effect of EK supplementation on cognitive function. The remaining studies failed to report complete results (i.e., pre-post intervention), or presented the results as median.

**Figure 3:**
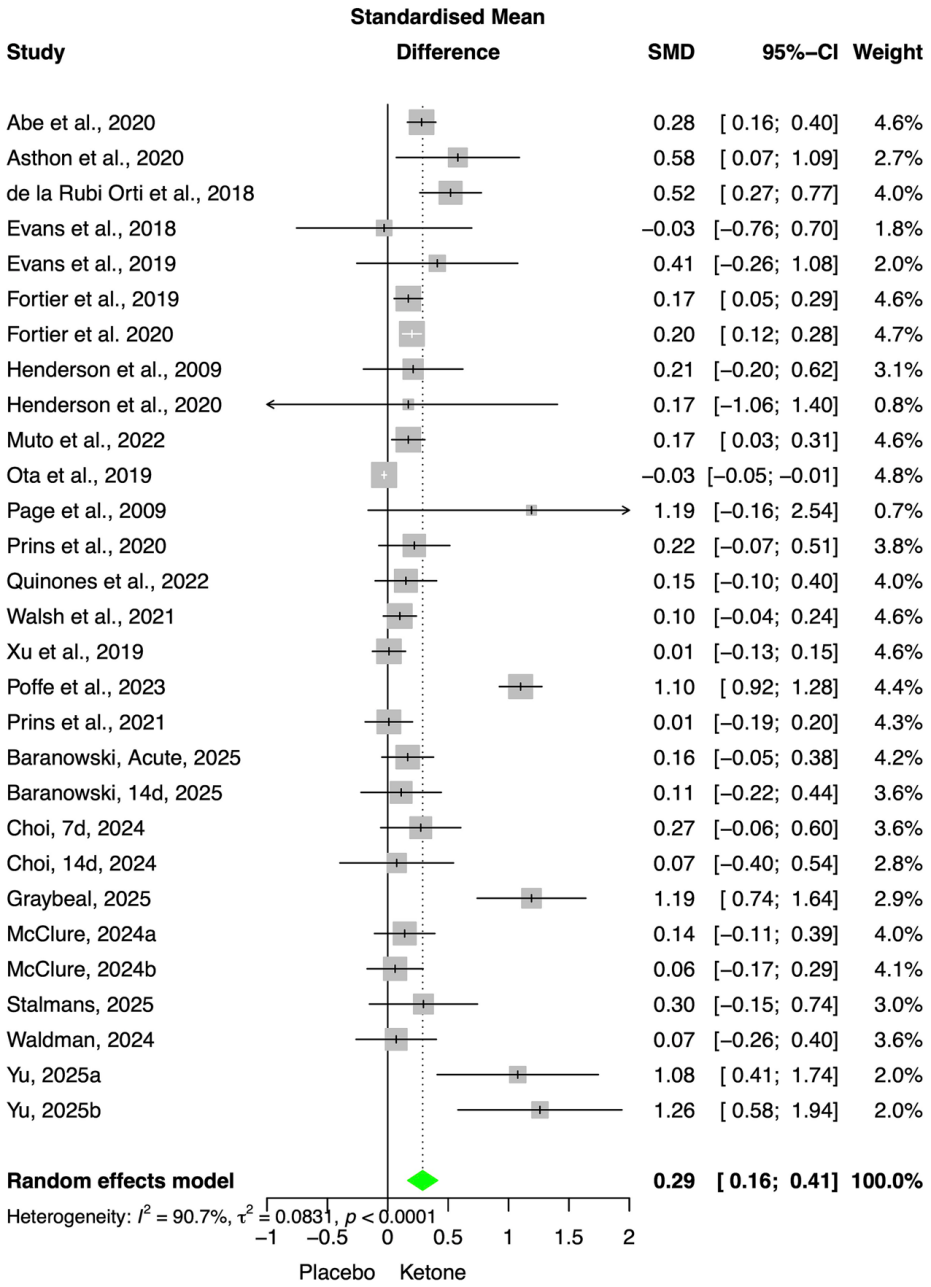
Overall meta-analysis for included protocols.

First, we assessed the overall effect of EK on cognitive function, pooling results from 29 protocols. The overall pooled effect was positive and statistically significant (SMD = 0.29, [95% CI 0.16–0.41], *p* < 0.001); using random effect model due to high heterogeneity (I^2^ = 91%, *p* < 0.001; Figure 3). Risk of bias was assessed using funnel plot and Egger’s regression and did not show asymmetry [Egger’s intercept = 2.65 (0.89), *p* = 0.08, Supplementary Figure 1]. The sensitivity analysis did not highlight the presence of outliers or studies with implausible results (extremely large effect; Supplementary Figure 2).

Next, we analyzed the differences between studies assessing the long-term (> 13 days) and immediate effects of EK. While no statistically significant difference between the duration groups (*p* = 0.11) was found, there is a tendency for a larger effect for studies assessing the immediate effect (SMD = 0.39 [0.16–0.62], *p* < 0.001) in comparison with longer studies (SMD = 0.19 [0.12–0.26], *p* < 0.001; Supplementary Figure 3).

When comparing the type of supplementation, the two most common supplement types, KetE and MCT, were studied. We did not find statistically significant differences between KetE and MCT (*p* = 0.083; Supplementary Figure 4), but a tendency for an overall larger positive effect on cognition for KetE (SMD = 0.37 [0.14–0.60], *p* = 0.004) in comparison with MCT (SMD = 0.15 [0.06–0.25], *p* = 0.018). As only two studies used a combination of KD and MCT, one study described the combination of MCT and BHB salt and one with coconut oil, there were not included in this analysis.

We then compared the cognitive effects of EK in studies of subjects with, and without the most included pathologies, MCI and AD. We expected that EK might provide greater benefit to subjects with a pathology that compromised their cognitive function. Surprisingly, we did not find a statistically significant difference (*p* = 0.077; Supplementary Figure 5) between the effect on healthy subjects (SMD = 0.36 [0.18–0.54], *p* = 0.004) and between subject with MCI or AD (SMD = 0.15 [0.01–0.30], *p* = 0.048).

We were interested to explore if the presence of a stressor such as exercise would modulate any effect of EK on cognition. No statistically significant differences (*p* = 0.89; Supplementary Figure 6) were found between studies using no stressor (SMD = 0.28 [0.15–0.42], *p* < 0.001) and studies that included an exercise-based stress (SMD = 0.26 [−0.03–0.55], *p* = 0.095).

**Figure 4:**
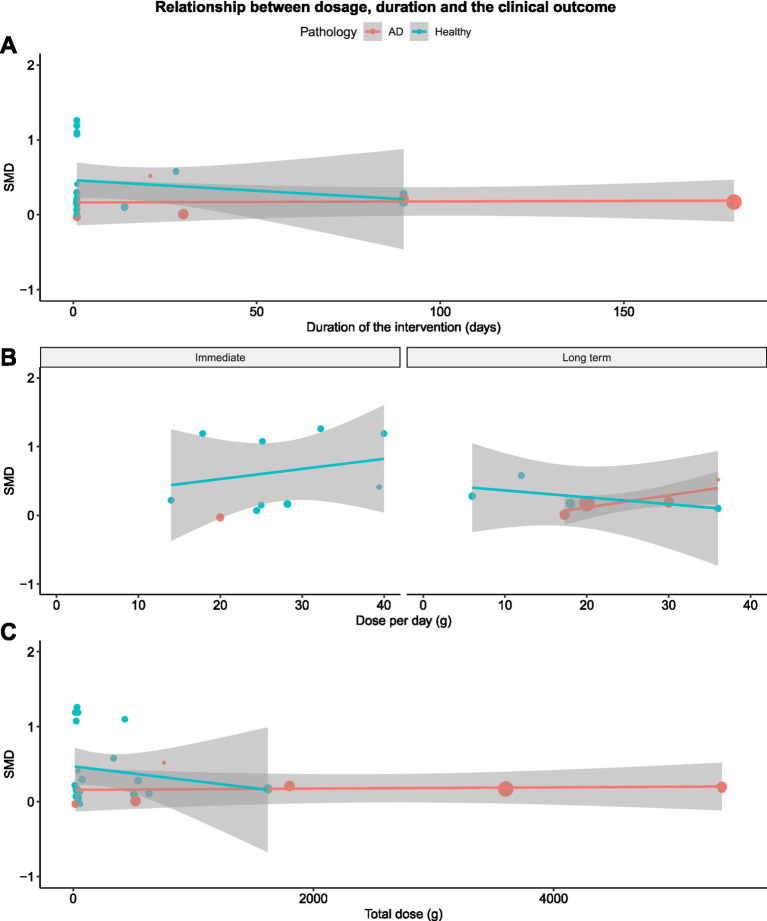
Meta-regression for the effect of duration (Panel **A**), dose per day (Panel **B**), and total dose (Panel **C**) on cognition.

Next, we performed meta-regression analysis to assess the relationship between cognitive outcomes and variables of interest. Firstly, we used meta-regression to determine if the duration of the EK intervention was related to cognitive outcomes. We did not find a significant relationship between the total duration of supplementation and cognitive outcomes for the overall dataset [*β* = −0.0011 (SE = 0.0012), *p* = 0.36; Figure 4A]. However, when analyzed by subgroups, the data showed a tendency toward a positive effect of longer interventions for MCT supplementation, although this effect was not statistically significant [*β* = 0.001 (0.0006), *p* = 0.089], see Table 3 for complete results.

**Table 3:** Meta-regression between the effect of ketone bodies supplementation on cognitive function for the total dose, the dose by day and the total duration of the supplementation.

Conditions	Duration		Dose by day		Total dose	
	β (SE)	*p*-value	β (SE)	*p*-value	β (SE)	*p*-value
Total	−0.0011 (0.0012)	0.36	**0.0020 (0.0005)**	**<0.001**	0.0001 (0.0001)	0.42
MCT	0.001 (0.0006)	0.089	−0.0016 (0.0069)	0.82	0.0001 (0.0001)	0.58
KetE	−0.0261 (0.0250)	0.29	0.0017 (0.001)	0.073	−0.0001 (0.0006)	0.86
Healthy	−0.0020 (0.0032)	0.53	**0.002 (0.0005)**	**<0.001**	0.0001 (0.0002)	0.42
AD	0.0004 (0.001)	0.68	**0.0231 (0.0032)**	**<0.001**	0.0001 (0.0001)	0.68

Boldfaced values indicate statistical significance. SE, standard deviation.

Secondly, we examined how the dose of EK consumed influenced the observed cognitive outcomes for the overall dataset. While a greater total EK dose over the study period did not significantly correlate with additional cognitive improvements [*β* = 0.0001 (0.0001), *p* = 0.42, Figure 4C], increasing daily dose of EK was strongly related to greater improvement in cognitive function across all studies [*β* = 0.0020 (0.0005), *p* < 0.001; Figure 4B]. We then repeated the meta-regression to determine if the relationship between daily dose against and outcomes persisted in further subgroup analyses. The relationship between greater daily dose and cognitive improvements remained significant for both healthy participants [*β* = 0.0022 (0.0005), *p* < 0.001] and those with AD [*β* = 0.0231 (0.0032), *p* < 0.001]. These findings imply that higher daily doses of EK consistently result in greater cognitive improvements across different populations and supplement types.

## Discussion

The aim of this systematic review and meta-analysis was to determine the effect of EK supplementation on cognitive function in humans. Across the included studies, EK was associated with modest but statistically significant improvements in cognitive performance, with evidence of efficacy in both healthy adults and those with AD and MCI. Overall, these findings highlight the promise of exogenous ketones as a novel strategy to target cognitive function, while also highlighting key knowledge gaps that must be addressed to inform clinical translation.

The positive effect of EK on cognition demonstrated by our overall, pooled analysis is in alignment with the primary teleological function of ketones as a back-up substrate for the brain during starvation (4). However, considering the context of cerebral substrate provision during fasting (i.e., low carbohydrate availability) compared to in the studies included here (i.e., EK alongside sufficient carbohydrate) it is, in fact, perhaps surprising that cognitive function following EK exceeded function under conditions of normal, ample substrate availability. This observation hints at the importance of mechanisms beyond simple substrate oxidation to the functional effect of EK. Supporting this interpretation, a large observational study found a cognitive benefit of BHB concentrations over as low as ~0.3 mM, at which oxidation would likely be low-to-minimal (30). Experimental and clinical studies have further suggested that EK may act to increase cerebral blood flow (7, 8), or increase brain network stability (47) and neurotransmitter release (10, 11). Taken together, these findings support a model in which ketones function as not only a metabolic substrate, but also as signaling metabolites (48) with distinct physiological effects that may contribute to enhanced cognitive function.

The temporal dynamics of cognitive responses to EK provide additional insight into potential mechanisms. While no statistically significant difference was found between studies assessing immediate versus long-term effects, the results approached the threshold for significance (*p* = 0.11), suggesting potential heterogeneity in response across study durations. However, meta-regression analysis did not identify intervention duration as a critical determinant of cognitive outcomes, with only a non-significant trend observed within the MCT subgroup. Interpretation of duration effects is further limited by the small number of long-term studies with KetE. It is possible that cognitive effects of exogenous ketones may, in some contexts, reflect mechanisms beyond their acute role as an oxidative substrate. If acute elevations in BHB acting primarily as an oxidative substrate were the dominant mechanism, cognitive effects might be expected to coincide closely with periods of elevated circulating ketones. At present, the latency of onset and persistence of cognitive effects following EK consumption is unknown; underscoring the need for studies explicitly designed to characterize these temporal features.

Beyond timing, both supplement type and dose appear to be important determinants of cognitive response. Both KetE and MCT were associated with statistically significant cognitive improvement, although larger effect sizes were observed for KetE. Meta regression analysis further indicated that higher daily ketone dose may be required to elicit measurable effects. Direct evidence supporting this relationship comes from the study by Fortier et al., in which greater circulating ketone concentrations were associated with a greater cognitive improvement during long-term MCT supplementation (25). Similar dose-dependent relationships between concentration and functional outcomes have been reported in other physiological systems, including the cardiovascular system where greater ketone availability has been linked to larger increases in cardiac output (49). Given that KetE have a larger impact on circulating ketones concentrations than both MCT and ketone salts (22, 23), their use in long duration studies of cognition represents a promising direction for future research. However, the most ketogenic and most widely studied ketone ester, (R)-3-hydroxybutyl (R)-3-hydroxybutyrate, is known to have a bitter taste which may limit long-term use, whereas MCTs and medium chain fatty acid esters such as bis-ocatnoyl (R),1-3-butanediol have a neutral taste, making them more translatable for long-term use, despite a lower ketone exposure. These trade-offs highlight the need to balance biological potency with translational practicality.

Several limitations constrain the generalizability of these findings. Most notably, substantial heterogeneity was observed across studies with respect to participant population, interventions (type of EK, dosage, duration), and outcome measures. This high heterogeneity (I^2^ = 91%) limits the extent to which pooled estimates can be generalized. Additionally, many included studies employed short interventions, and there remains a relative paucity of long-term studies and long-term follow up, particularly those using KME. The lack of standardized cognitive endpoints and limited long-term follow-up further restrict conclusions regarding the durability and clinical significance of observed effects. These limitations emphasize the need for more harmonized study designs in future investigations.

Despite these limitations, the present findings are promising and offer interesting perspective for future research and clinical translation. Given the beneficial cognitive effects of EK in both healthy and cognitively impaired individuals, no known safety concerns and acceptable tolerance profiles, EK are a candidate for further research and clinical translation. It is likely that the greatest effect would be seen if EK were used in synergy with other interventions, such as diet and exercise; this should be addressed in future research. No studies have addressed if EK can play a role in prevention, this too is an avenue of potential application. Other key questions highlighted by this analysis and foundational to the field of EK application at large, include the choice of EK supplement type (MCT vs. KetE), dose selection and possible drivers of individual differences between individuals including metabolic responsiveness, cognitive and physical health.

In conclusion, this systematic review and meta-analysis provides quantitative evidence that exogenous ketone supplementation is associated with modest but significant improvements in cognitive performance across a range of populations. These effects appear to be consistent across study contexts and are not restricted to specific disease states or acute metabolic stressors. While substantial heterogeneity and limited long-term data temper definitive conclusions, the findings support further investigation into EK as a metabolic strategy to support cognitive health. Continued mechanistic and clinical research will be essential to define optimal formulations, dosing strategies, and use cases for exogenous ketones in cognitive function and cognitive aging.

## Data Availability

The data can be accessed by contacting the authors of the original studies. Requests to access these datasets should be directed to BS, bstubbs@buckinstitute.org.

## Supplementary material

SUPPLEMENTARY FIGURE 1 Funnel plot assessing publication bias across studies of ketone supplementation. Each point represents an individual study plotted by effect size (Hedges’ *g*) against standard error. Dashed lines indicate significance thresholds (*p* < 0.05, *p* < 0.025, *p* < 0.01). Asymmetry in the funnel may indicate publication bias or small-study effects.

SUPPLEMENTARY FIGURE 2 Leave-one-out sensitivity analysis for the effect of ketone supplementation on cognition. Each row shows the pooled SMD and 95% CI after sequentially omitting one study. The overall estimate remained stable across all iterations (SMD range: 0.23 -0.32), with consistently high heterogeneity (I^²^ ≈ 83 -91%), indicating that no single study unduly influenced the pooled result.

SUPPLEMENTARY FIGURE 3 Eorest plot of the effect of ketone supplementation on cognitive performance, stratified by intervention duration (immediate vs. long-term). Standardised mean differences (SMDs) and 95% confidence intervals (CIs) are shown for each study, stratified by whether the intervention was administered as a single acute dose (Effect = Immediate) or over more than 13 days (Effect = Long term). The overall pooled random effects estimate favored ketone supplementation (SMD = 0.29, 95% CI [0.16; 0.41]). Effect sizes trended larger for immediate interventions (SMD = 0.39, 95% CI [0.16; 0.62]) compared to long-term interventions (SMD = 0.19, 95% CI [0.12; 0.26]), though subgroup differences were not statistically significant (p = 0.111). Substantial heterogeneity was observed overall (I^²^ = 90.7%, *p* < 0.0001) and within the immediate subgroup (I^²^ = 93.2%), while heterogeneity was lower within the long-term subgroup (I^²^ = 40.4%). SMD, standardised mean difference; CI, confidence interval.

SUPPLEMENTARY FIGURE 4 Forest plot of the effect of ketone supplementation on cognition, stratified by supplement type (MCT vs. ketone ester). SMDs and 95% CIs are shown per study and pooled separately for MCT (SMD = 0.15) and ketone ester (KE; SMD = 0.37) subgroups. No significant subgroup difference was detected (*p* = 0.0834). Substantial heterogeneity was present in both subgroups (I^²^ = 86.5% and 89.7%, respectively).

SUPPLEMENTARY FIGURE 5 Forest plot of the effect of ketone supplementation on cognition, stratified by participant health status (healthy vs. Alzheimer’s disease). SMDs and 95% CIs are shown per study and pooled separately for healthy participants (SMD = 0.36) and those with Alzheimer’s disease (AD; SMD = 0.15). Subgroup differences were not statistically significant (*p* = 0.0771), though effect sizes trended larger in healthy populations.

SUPPLEMENTARY FIGURE 6 Forest plot of the effect of ketone supplementation on cognitive performance, stratified by stressor condition (none vs. exercise). Standardised mean differences (SMDs) and 95% confidence intervals (CIs) are shown for each study, stratified by whether cognitive testing was administered at rest (Stressor = None) or following exercise (Stressor = Exercise). The overall pooled random effects estimate favored ketone supplementation (SMD = 0.29, 95% CI [0.16, 0.42]). Effect sizes were comparable between the no-stressor (SMD = 0.28) and exercise-stressor (SMD = 0.26) subgroups, with no significant difference between them (p = 0.897). Substantial heterogeneity was observed overall (I^2^ = 91.6%, p < 0.0001) and within both subgroups. SMD, standardised mean difference; CI, confidence interval.
