# Supplementary figures and images for: The effect of exogenous ketone bodies on cognition across health and disease: a systematic review and meta-analysis
Source: Front Nutr. 2026 Apr 15;13:1802531. doi: 10.3389/fnut.2026.1802531 (PMC13127162; doi:10.3389/fnut.2026.1802531)

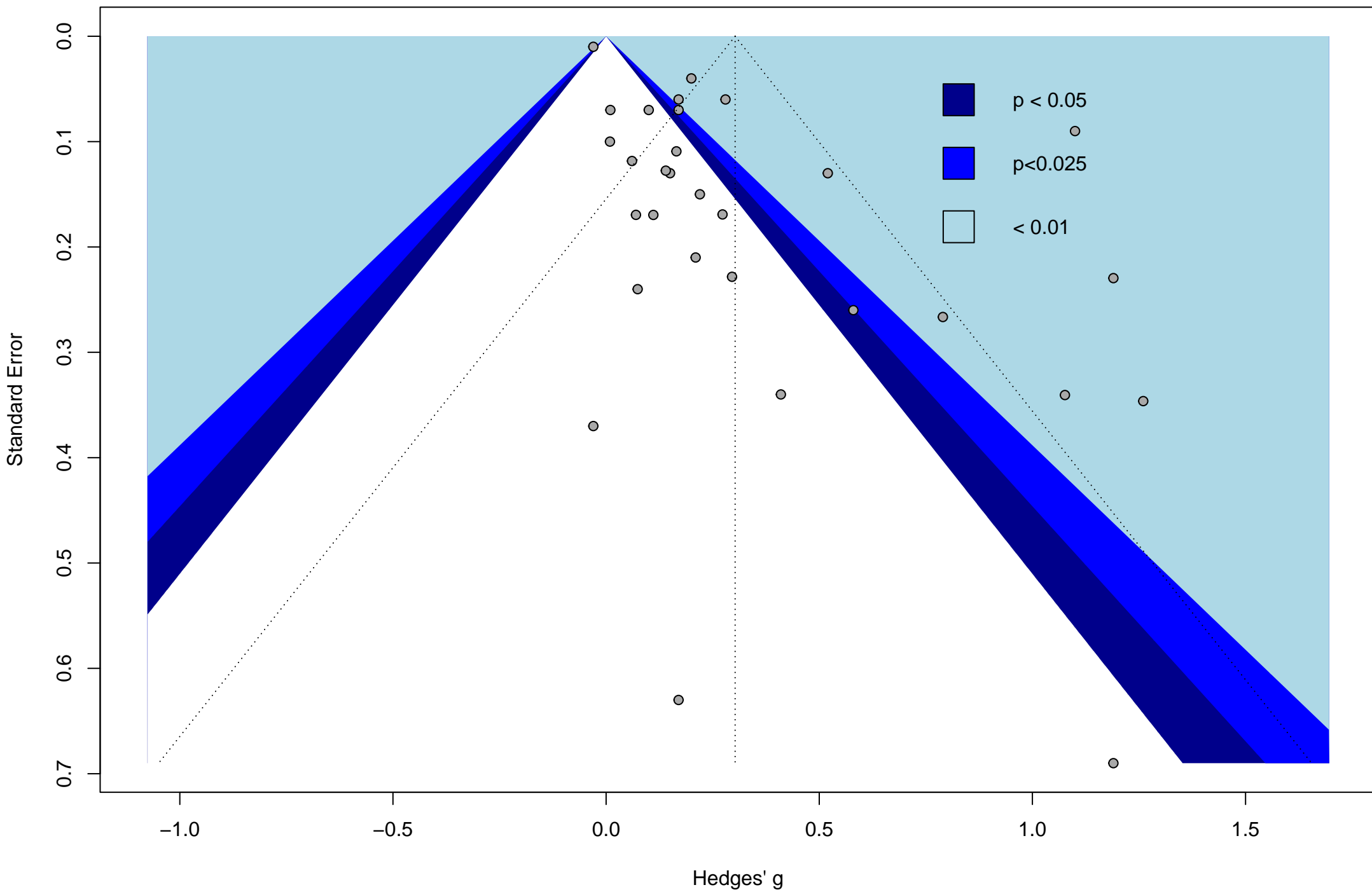

Supplement: SUPPLEMENTARY FIGURE 1 — Funnel plot assessing publication bias across studies of ketone supplementation. Each point represents an individual study plotted by effect size (Hedges’ g) against standard error. Dashed lines indicate significance thresholds (p < 0.05, p < 0.025, p < 0.01). Asymmetry in the funnel may indicate publication bias or small-study effects. [file Image_1.pdf]

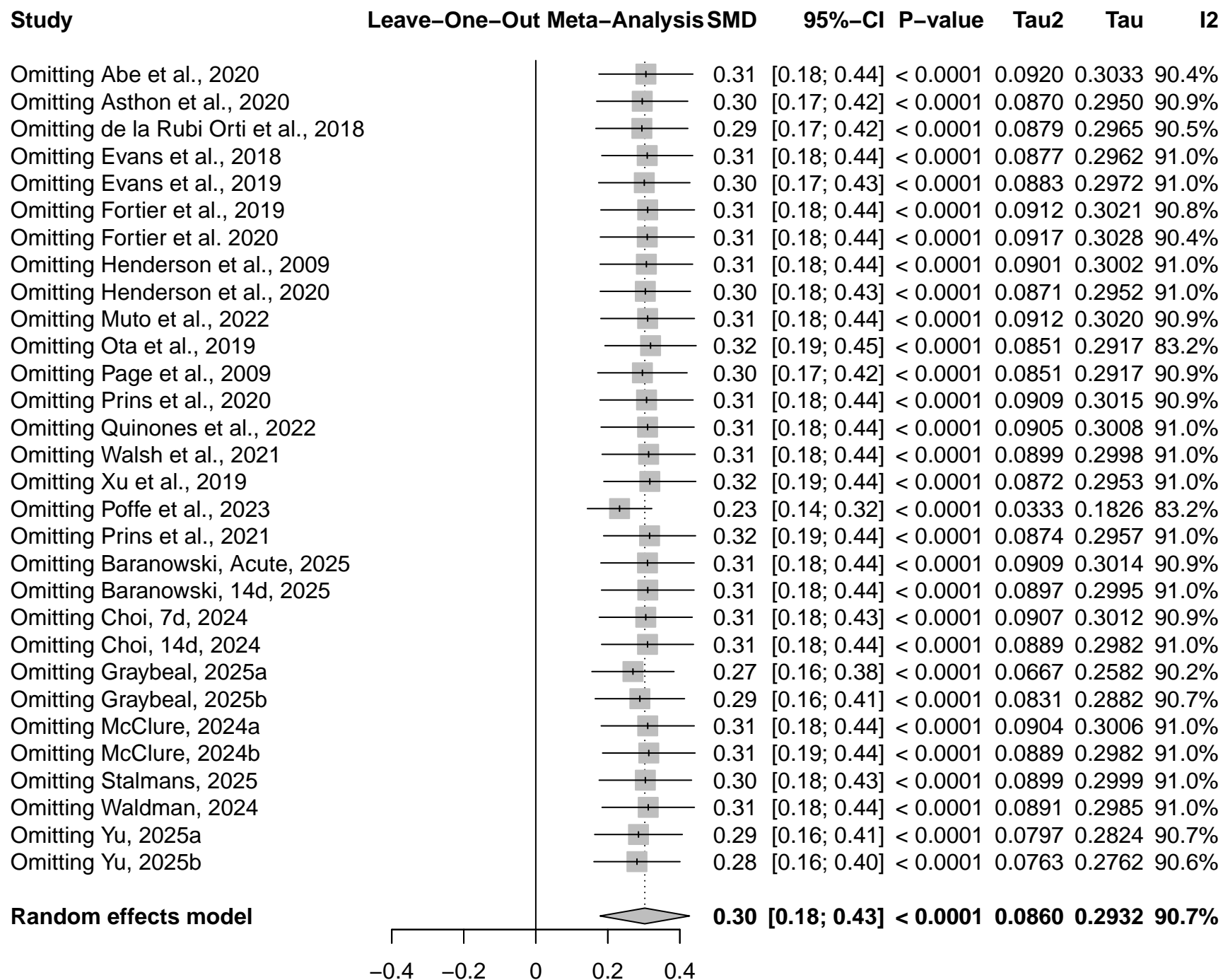

Supplement: SUPPLEMENTARY FIGURE 2 — Leave-one-out sensitivity analysis for the effect of ketone supplementation on cognition. Each row shows the pooled SMD and 95% CI after sequentially omitting one study. The overall estimate remained stable across all iterations (SMD range: 0.23 -0.32), with consistently high heterogeneity (I² ≈ 83 -91%), indicating that no single study unduly influenced the pooled result. [file Image_2.pdf]

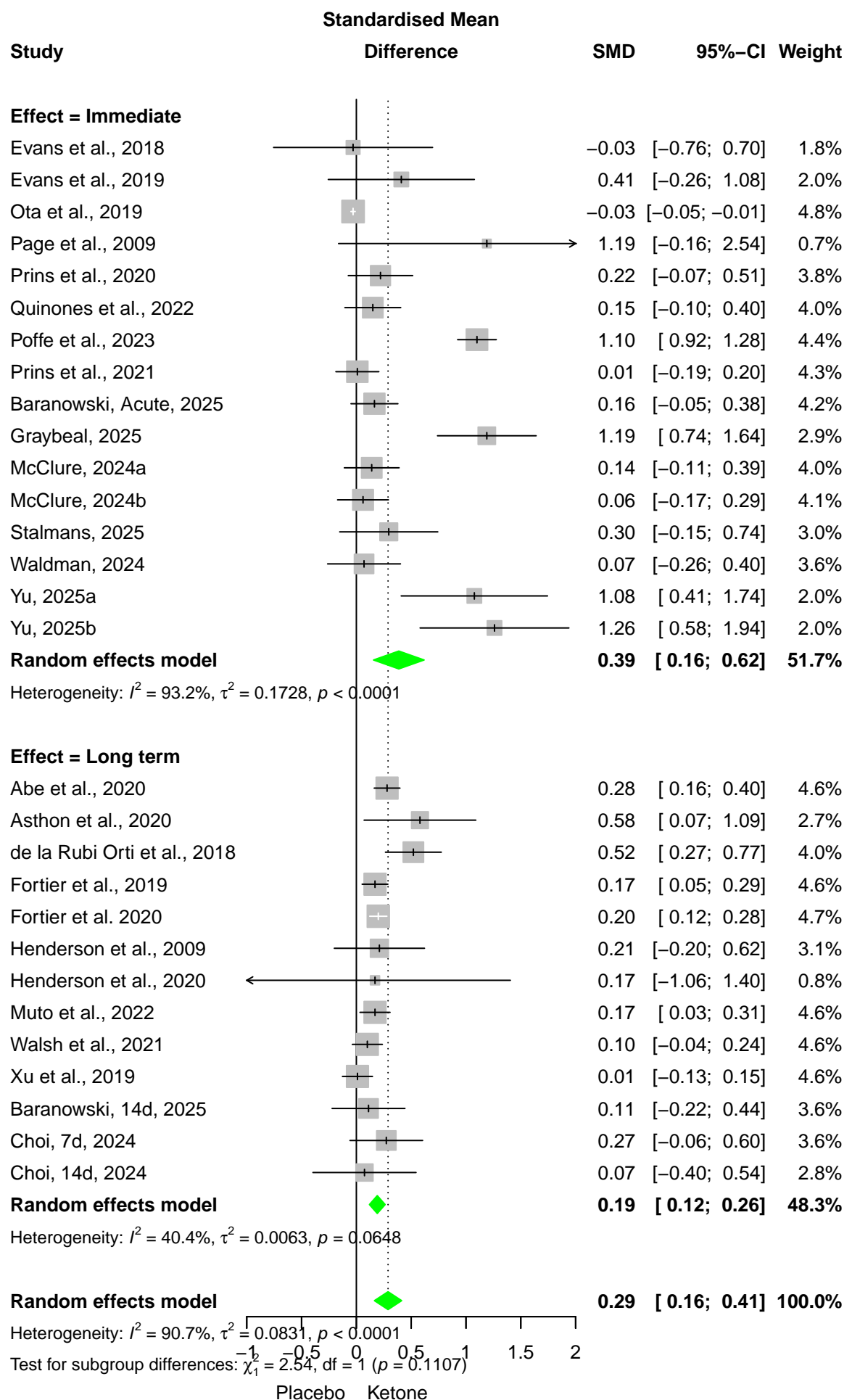

Supplement: SUPPLEMENTARY FIGURE 3 — Eorest plot of the effect of ketone supplementation on cognitive performance, stratified by intervention duration (immediate vs. long-term). Standardised mean differences (SMDs) and 95% confidence intervals (CIs) are shown for each study, stratified by whether the intervention was administered as a single acute dose (Effect = Immediate) or over more than 13 days (Effect = Long term). The overall pooled random effects estimate favored ketone supplementation (SMD = 0.29, 95% CI [0.16; 0.41]). Effect sizes trended larger for immediate interventions (SMD = 0.39, 95% CI [0.16; 0.62]) compared to long-term interventions (SMD = 0.19, 95% CI [0.12; 0.26]), though subgroup differences were not statistically significant (p = 0.111). Substantial heterogeneity was observed overall (I² = 90.7%, p < 0.0001) and within the immediate subgroup (I² = 93.2%), while heterogeneity was lower within the long-term subgroup (I² = 40.4%). SMD, standardised mean difference; CI, confidence interval. [file Image_3.pdf]

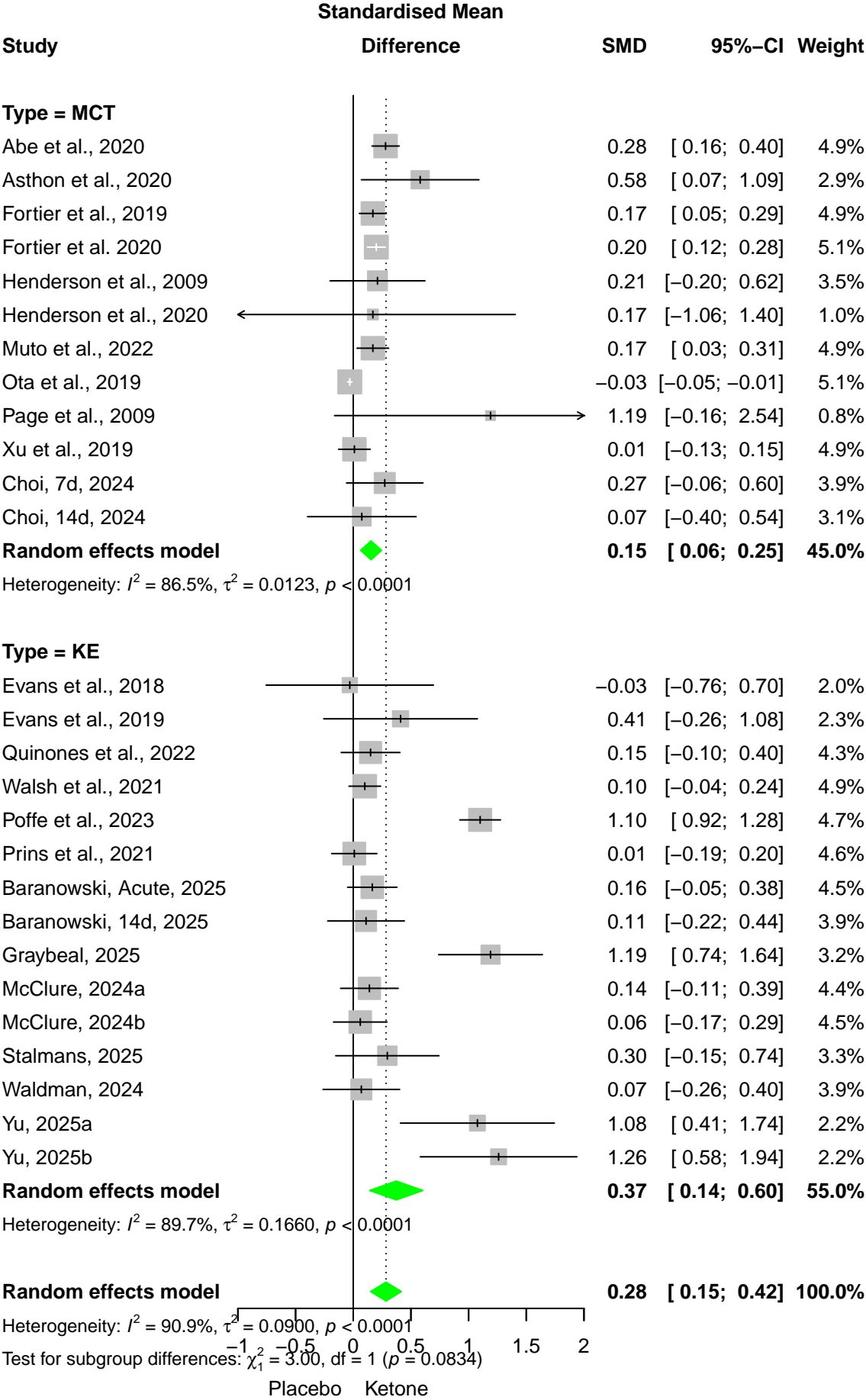

Supplement: SUPPLEMENTARY FIGURE 4 — Forest plot of the effect of ketone supplementation on cognition, stratified by supplement type (MCT vs. ketone ester). SMDs and 95% CIs are shown per study and pooled separately for MCT (SMD = 0.15) and ketone ester (KE; SMD = 0.37) subgroups. No significant subgroup difference was detected (p = 0.0834). Substantial heterogeneity was present in both subgroups (I² = 86.5% and 89.7%, respectively). [file Image_4.pdf]

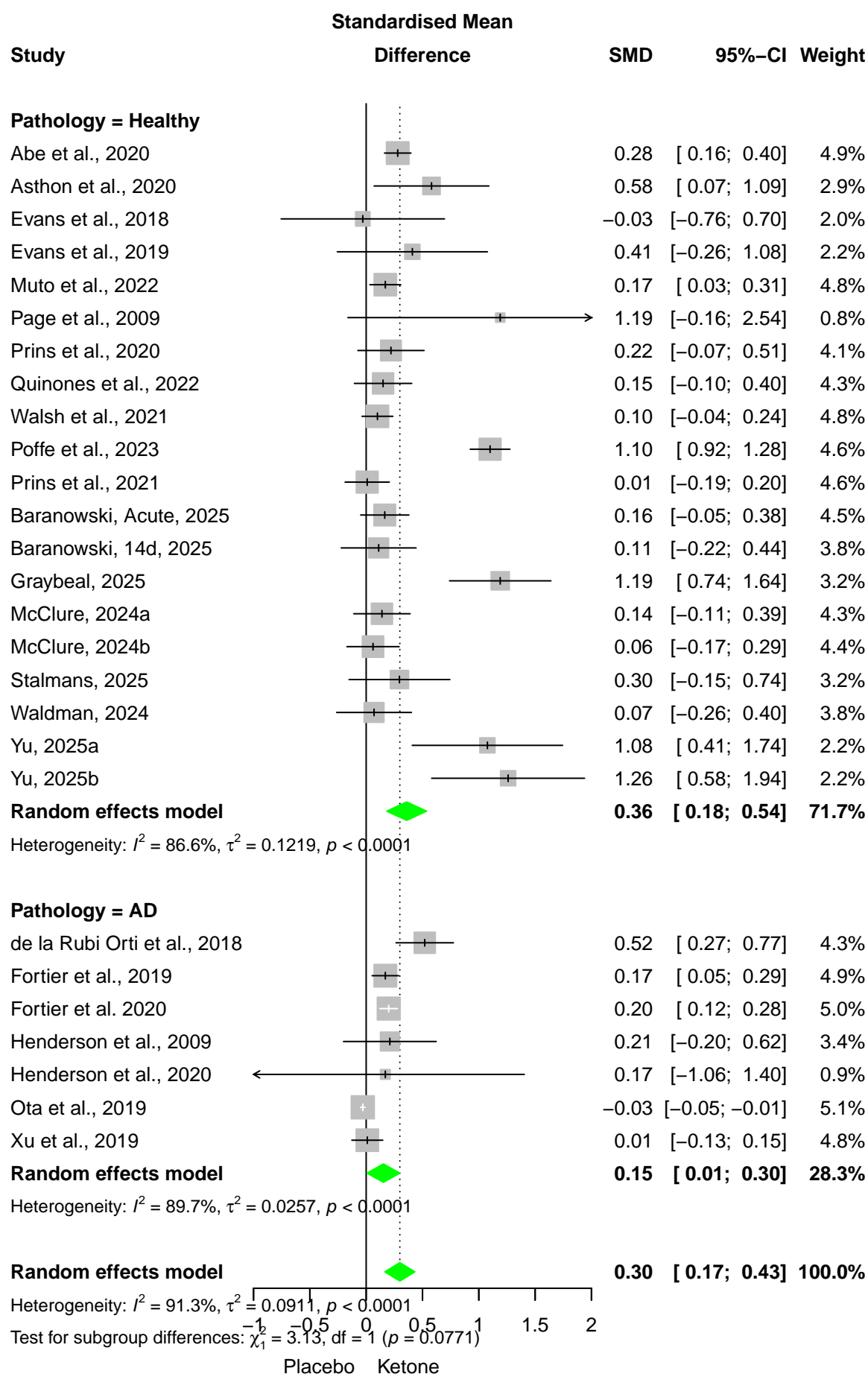

Supplement: SUPPLEMENTARY FIGURE 5 — Forest plot of the effect of ketone supplementation on cognition, stratified by participant health status (healthy vs. Alzheimer’s disease). SMDs and 95% CIs are shown per study and pooled separately for healthy participants (SMD = 0.36) and those with Alzheimer’s disease (AD; SMD = 0.15). Subgroup differences were not statistically significant (p = 0.0771), though effect sizes trended larger in healthy populations. [file Image_5.pdf]

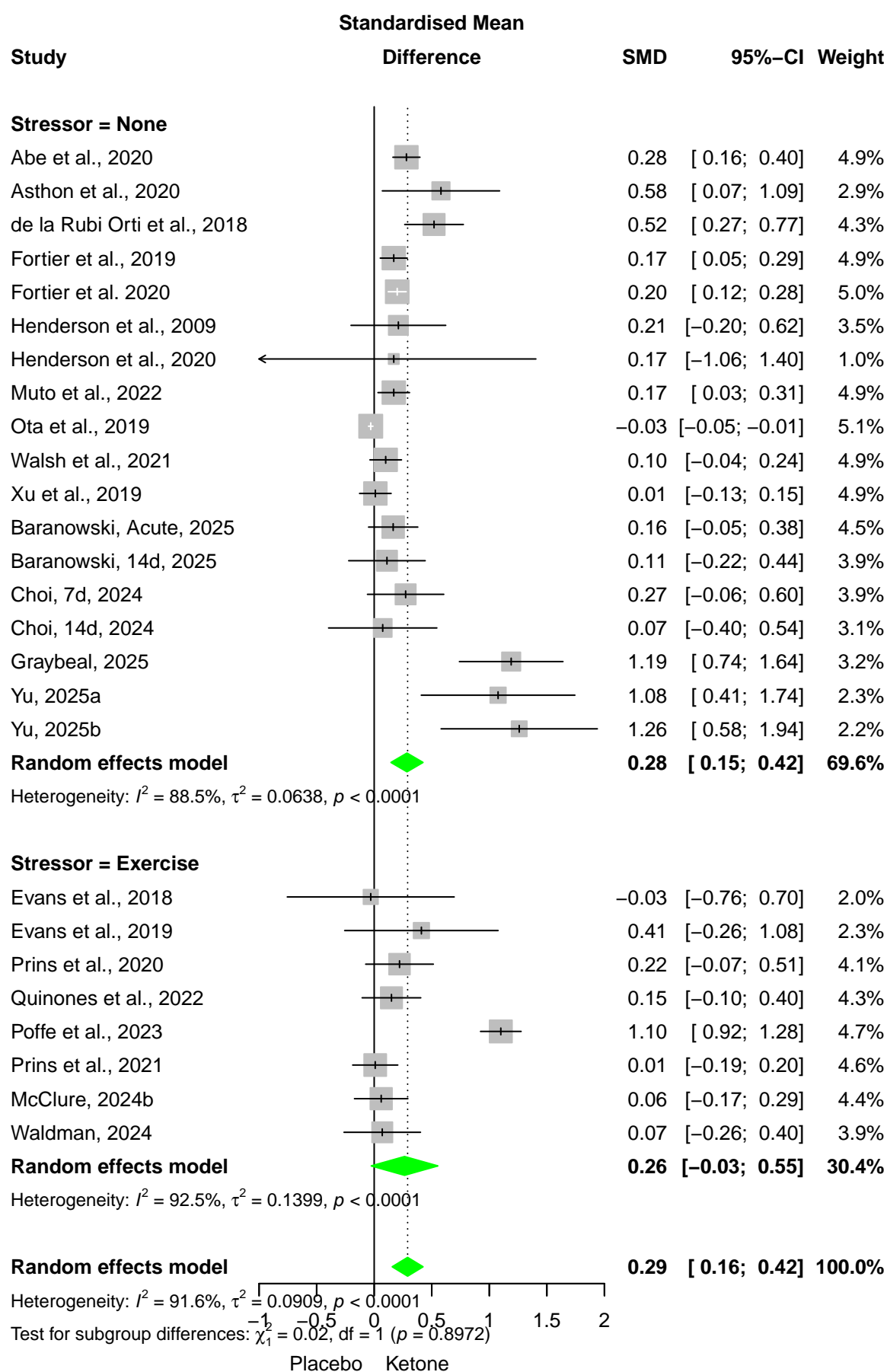

Supplement: SUPPLEMENTARY FIGURE 6 — Forest plot of the effect of ketone supplementation on cognitive performance, stratified by stressor condition (none vs. exercise). Standardised mean differences (SMDs) and 95% confidence intervals (CIs) are shown for each study, stratified by whether cognitive testing was administered at rest (Stressor = None) or following exercise (Stressor = Exercise). The overall pooled random effects estimate favored ketone supplementation (SMD = 0.29, 95% CI [0.16, 0.42]). Effect sizes were comparable between the no-stressor (SMD = 0.28) and exercise-stressor (SMD = 0.26) subgroups, with no significant difference between them (p = 0.897). Substantial heterogeneity was observed overall (I2 = 91.6%, p < 0.0001) and within both subgroups. SMD, standardised mean difference; CI, confidence interval. [file Image_6.pdf]
